# Research Roadmap for Tuberculosis Transmission Science: Where Do We Go From Here and How Will We Know When We’re There?

**DOI:** 10.1093/infdis/jix353

**Published:** 2017-11-03

**Authors:** Sara C Auld, Anne G Kasmar, David W Dowdy, Barun Mathema, Neel R Gandhi, Gavin J Churchyard, Roxana Rustomjee, N Sarita Shah

**Affiliations:** 1 School of Medicine and Rollins School of Public Health, Emory University, and; 2 Division of Global HIV and Tuberculosis, Centers for Disease Control and Prevention, Atlanta, Georgia;; 3 Bill and Melinda Gates Foundation, Seattle, Washington;; 4 Bloomberg School of Public Health, Johns Hopkins University, Baltimore, and; 5 Division of AIDS, National Institute of Allergy and Infectious Diseases, National Institutes of Health, Rockville, Maryland;; 6 Department of Epidemiology, Mailman School of Public Health, Columbia University, New York, New York;; 7 Aurum Institute and the School of Public Health, University of Witwatersrand and; 8 Advancing Care for tuberculosis and HIV, South African Medical Research Council, Johannesburg, South Africa

**Keywords:** tuberculosis, transmission, public health

## Abstract

High rates of tuberculosis transmission are driving the ongoing global tuberculosis epidemic, and there is a pressing need for research focused on understanding and, ultimately, halting transmission. The ongoing tuberculosis–human immunodeficiency virus (HIV) coepidemic and rising rates of drug-resistant tuberculosis in parts of the world add further urgency to this work. Success in this research will require a concerted, multidisciplinary effort on the part of tuberculosis scientists, clinicians, programs, and funders and must span the research spectrum from biomedical sciences to the social sciences, public health, epidemiology, cost-effectiveness analyses, and operations research. Heterogeneity of tuberculosis disease, both among individual patients and among communities, poses a substantial challenge to efforts to interrupt transmission. As such, it is likely that effective interventions to stop transmission will require a combination of approaches that will vary across different epidemiologic settings. This research roadmap summarizes key gaps in our current understanding of transmission, as laid out in the preceding articles in this series. We also hope that it will be a call to action for the global tuberculosis community to make a sustained commitment to tuberculosis transmission science. Halting transmission today is an essential step on the path to end tuberculosis tomorrow.

## GETTING TO ZERO REQUIRES INTERRUPTING TRANSMISSION TODAY

With the release of the most recent Global Plan to Stop tuberculosis, the attention and efforts of the global tuberculosis community are rightly focused on the necessary goal to end tuberculosis [1]. Achieving this ambitious goal will not be easy. Tuberculosis, often called the oldest disease of mankind, is now the leading global killer among infectious diseases and the leading cause of death for people with HIV [2, 3]. Rising rates of drug resistance in parts of the world bring further urgency to the imperative to end tuberculosis because each case of drug resistance is orders of magnitude more difficult to treat with second- and third-line drug regimens that are more costly for tuberculosis programs, more toxic for patients, and often less effective [4]. The Global Plan calls for a paradigm shift in our approach to combating tuberculosis, including a change in mindset whereby tuberculosis has no place in our societies—with a push for more patient-centered care, programmatic innovations, and sustained increases in funding. Yet a critical component of what will be necessary for success has been too often neglected: an explicit focus on halting transmission.

The analogy of “turning off the tap” of new tuberculosis cases is often upheld as key for decreasing the global burden of tuberculosis [5]; yet our ability to stop new cases hinges directly on our capacity to halt transmission. As with so much of tuberculosis care, our current tools for halting transmission have been around for decades: active case finding, contact investigations, and targeted testing and treatment of latent tuberculosis infection. These interventions have proven quite successful in decreasing the tuberculosis burden at the population level in key historical trials [6–8]. However, as illustrated by several large-scale community-based studies, implementation of these measures in modern high-burden settings is often challenging—and, without substantial scale-up of existing interventions and the addition of novel tools, it is unlikely that we will achieve the goal of ending tuberculosis in those areas [9–12]. For example, in sub-Saharan Africa, transmission in the community is often so intense that household members and other known contacts only account for a minority of transmission events [13–17]. As a result, contact investigation is unlikely to halt tuberculosis transmission on its own in higher-burden contexts, even though it may still avert a high absolute number of transmission events. Additional gaps in our understanding of the biology and local determinants of transmission underscore just how little we know about transmission—and how that ignorance may be undermining our efforts to end tuberculosis.

Although others have advocated for halting transmission [18–22], we believe that this series offers a necessary perspective by focusing on transmission as a unique scientific discipline. While we must continue to deploy tools that are currently available and maximize the benefit from interventions that are known to be effective, we must also mobilize to develop new tools for combating transmission—new tools to understand where, how, and between whom transmission is happening. A roadmap for tuberculosis transmission research will enable us to track the most efficient and effective pathways for studying, understanding, and, ultimately, halting tuberculosis transmission. Only by focusing on transmission as both a sentinel event and an outcome of interest can we become proactive, rather than reactive, to new cases of tuberculosis. The roadmap herein draws from the articles in this series to chart an initial research path across the spectrum of basic sciences to applied and operations research. We hope that this roadmap provides tuberculosis researchers, programmers, policy makers, funders, and advocates with a forecast of research needs and a means for charting progress against milestones. Advances in transmission science will provide us with the necessary means for interrupting transmission wherever it may be occurring. Only then will we be able to design effective biomedical and sociobehavioral interventions to truly turn off the tap.

## CURRENT TRANSMISSION RESEARCH NEEDS

In this section, we review and highlight some of the key points from the 3 scientific articles in this series. Within each research area—infectiousness and susceptibility, drivers of tuberculosis transmission, and interventions to halt the transmission of tuberculosis—we have emphasized gaps in our knowledge of transmission science that present immediate opportunities for high-impact research.

### Infectiousness and Susceptibility

Multiple gaps remain in our understanding of source case infectiousness, aerosolization and airborne survival of *Mycobacterium tuberculosis* (Mtb), and host susceptibility. Source case infectiousness may be influenced by a number of individual-level factors, including disease site, sex, ethnicity, HIV status, and social risk factors [23–25]. Yet our ability to account for transmission being driven by each of these factors is limited. Furthermore, it is not yet known how subclinical disease or fluctuations in respiratory symptoms might contribute to transmission [26, 27]; nor is it known how variability in respiratory mechanics, tidal breathing, nocturnal breathing, or cough impact the likelihood of producing droplet nuclei with viable Mtb [28–30].

Although it is possible that pathogen-level characteristics, such as organism phenotype or lineage, might affect the aerobiology of tuberculosis, the role of shear forces and air and fluid dynamics is also likely to be quite important for aerosol transmission [29]. Once Mtb is released into the air, it remains unclear what proportion of droplet nuclei contain viable bacilli, how long those bacilli remain viable in the open environment, and whether there are differences between aerosolized versus alveolar Mtb [31, 32]. Elucidating the nature of tuberculosis transmission events by studying patient aerosols, also known as aerobiology, will address critical questions about the physiology and metabolic state of aerosolized Mtb, the viability and persistence of infectious aerosols, and the key characteristics associated with infectiousness.

Once Mtb is aerosolized in an indoor environment, multiple infection control interventions that reduce exposure and inhalation (“environmental controls”) have been shown to be effective, including natural or mechanical ventilation and upper room ultraviolet germicidal irradiation [33]. Reducing crowding in hospital and clinic waiting areas will further limit the effective contact rate between infectious persons and potentially susceptible contacts [34]. Yet, cost of installation, logistics of maintenance, and practical considerations (eg, climate that precludes opening doors and windows) often present barriers to implementation of environmental controls. Innovations are needed that simplify and reduce costs of healthcare facility redesign, with monitoring of airborne infectious particles, alerts when they above a critical level, and direct “cleansing” of shared air.

The determinants behind the probability of aerosol deposition of Mtb in a susceptible host are yet another unknown. There are a number of recent efforts to characterize cough aerosol production and exposure, but there is no current standard for determining whether a particular individual was exposed to Mtb. For close contacts of tuberculosis patients with a negative tuberculin skin test (TST) or interferon-gamma release assay (IGRA), there is no way to know whether they were indeed “adequately” exposed to Mtb, whether they had successful “early clearance” without mounting a T-cell–mediated adaptive immune response, or whether they are simply anergic with a false-negative TST or IGRA. This inability to characterize and quantify recent exposure, including among animal models, poses a substantial impediment to studies of transmission and transmissibility. Efforts to offset host vulnerability and enhance selective immune responses may prove critical for the design of a tuberculosis vaccine that is capable of preventing infection and not simply diminishing bacterial burden.

### Drivers of Tuberculosis Transmission—Know Your Epidemic, Know Your Intervention

Mounting evidence from high tuberculosis burden countries, with varying levels of HIV and drug-resistant tuberculosis, implicate ongoing transmission as the driving force maintaining high levels of tuberculosis incidence [17, 35, 36]. Drivers of transmission can be conceived of as 3 stages along a cascade of transmission: (1) contact between infectious and susceptible individuals; (2) infectiousness of a particular individual; and (3) susceptibility to disease of exposed individuals [22]. These stages are heterogeneous at regional and national levels, and the relative role of each stage is heavily dictated by local attributes, including access to health care, population age structure, housing, population density, and migration [37, 38]. Variations in individual host susceptibility, in line with the local prevalence of HIV, malnutrition and low body mass index, diabetes mellitus, tobacco use, alcoholism, and silicosis, can also dramatically alter the transmission cascade [39, 40]. The strength of local tuberculosis control programs’ case finding and treatment strategies is yet another factor that can affect local transmission dynamics; a larger proportion of undiagnosed or untreated patients will contribute to ongoing transmission in a community. This heterogeneity of transmission poses a substantial challenge to tuberculosis control. To design efficacious and efficient tuberculosis control strategies, it will be necessary to delineate the relative contribution of the various drivers of tuberculosis in different settings. Ultimately, drivers of transmission must be studied and understood on a local level.

At present, measuring levels of tuberculosis transmission is also exceedingly difficult. The natural history and long latency period of tuberculosis means that observed cases of disease reflect a mix of recent transmission and reactivation disease from a remote infection. In addition, most cases observed through passive case-finding systems underestimate the number of undiagnosed, but diagnosable, infectious individuals in the community. Current methods to measure transmission include examining case notification rates and trends in TST or IGRA. However, real-time, definitive diagnosis of recent exposure to Mtb at the individual level is not possible with any of the currently available tests (eg, TST, IGRA, chest X-ray, sputum analysis). Yet there are several recent reports of promising new approaches to identifying recent exposure, including a T-cell immune signature capable of differentiating recent from remote tuberculosis infection and a blood transcriptomic signature associated with greater risk of progression to active tuberculosis disease [41, 42]. Although these signatures need to be validated in other settings, this type of biomarker of recent exposure would enable precisely targeted preventive therapy (akin to ring prophylaxis) and accelerate identification of tuberculosis “hotspots”—areas of high tuberculosis incidence—for earlier and more effective implementation of infection control measures [43]. Such hotspot detection and elimination represents a stop-gap measure to interrupt transmission and reduce the global burden of tuberculosis while new drugs, diagnostics, and vaccines are progressing through the product development pipeline. These hotspot communities could also serve as priority areas for piloting new transmission interventions as they become available [44].

More recently, molecular epidemiology using whole genome sequencing has greatly facilitated studies of patient- and population-level transmission [13, 14]. Interestingly, in a number of studies that used genotyping in the late 1990s and early 2000s, cases with genetic links often did not have traditional epidemiologic links [16]. Several studies have aimed to estimate the proportion of transmission that occurs within households by comparing genotypes of cases occurring within households or community settings. These studies have found that in medium and high tuberculosis burden settings, the majority of tuberculosis transmission occurs outside of households or known close contacts [15, 45]. As such, although contact investigations remain a critical component of any effort to identify and eliminate tuberculosis in the community, the proportion of tuberculosis transmission that can be halted through contact investigations alone may be lower in some settings than traditionally anticipated [46]. Furthermore, the location where infections are occurring within these communities remains a major unknown in the transmission ecology of tuberculosis. Congregate locations that facilitate air exchange between infectious cases with noninfected individuals, such as in transport hubs, school classrooms, and prisons, have been implicated as hotspots of transmission and may offer opportunities for targeted interventions [47, 48]. However, it is unknown whether other, less obvious sites of congregation may also be driving ongoing transmission. An integrated approach that combines traditional epidemiology with methods drawn from spatial, demographic, network, and whole genome analysis will undoubtedly be required to identify such drivers.

### Interventions to Halt the Transmission of Tuberculosis

Ultimately, it is not simply enough to understand where and how tuberculosis transmission occurs; we must intervene to interrupt that transmission if we hope to have an impact on incidence. To be effective in this effort, it is critical to understand which interventions, implemented in what fashion, are most likely to avert the largest proportion of transmission events (specifically, those events that result in secondary cases of infectious tuberculosis) at the population level. For example, in settings where transmission originates largely from specific and identifiable populations (eg, young adult men [49]), it may be feasible to target case-finding efforts at those groups. Where reactivation is common in high-risk groups (eg, HIV-positive individuals [50], people with risk factors such as diabetes or malnutrition [51], elderly populations [52]), it may be critical to target preventive therapy to those populations. In congregate settings (eg, prisons or healthcare institutions), infection control and modification of the built environment can have a major impact in reducing transmission.

In prioritizing between different potential interventions in a given setting, creating “snapshots” of prevalent tuberculosis to understand who has active tuberculosis at a given time and how each case of infectious tuberculosis might have been averted can be helpful because these cases represent the tuberculosis transmission potential in a community. Novel study designs and statistical techniques (such as adaptive trials [53] and linkage of trial outcomes with transmission models [54]) can aid in developing an evidence base that speaks not just to the effectiveness of specific interventions but also to their comparative impact on transmission at the population level.

Although research to understand tuberculosis transmission is essential, we must link those efforts with rigorous evaluation of the efficacy and cost-effectiveness of transmission-halting interventions so that we can prioritize those activities most likely to prevent the greatest number of transmission events as rapidly as possible given available resources. Meanwhile, our armamentarium for halting tuberculosis transmission need not be limited to biomedical interventions. Historical data from Western Europe, where tuberculosis incidence declined 10% annually after World War II, tells us that socioeconomic development can reduce tuberculosis transmission. We should hypothesize broadly about how socioeconomic development may interrupt transmission and systematically test interventions for impact, including nutrition support and direct cash transfer programs to household or individuals in tuberculosis-affected communities [55]. An additional benefit of interventions aimed at reducing tuberculosis transmission through poverty alleviation is that they may also reduce the spread of emerging infectious diseases such as pandemic influenza and Ebola. Finally, when evaluations to halt transmission do not turn out as expected, as with the Thibela tuberculosis trial of isoniazid preventive therapy in South African gold mines [12], rigorous efforts must be undertaken to understand the reasons behind the negative findings so that any pitfalls can be adequately addressed or avoided in future studies [54].

### INTERRUPTING TRANSMISSION TO END TUBERCULOSIS

The global burden of tuberculosis cannot be understated. In addition to being the leading infectious disease killer and leading killer among people with HIV, the costs to healthcare systems, communities, families, and patients is unsustainable. For example, in Myanmar it is estimated that 65% of tuberculosis-affected households face catastrophic costs (ie, >20% of their annual household income) [3]. The World Health Assembly member states have endorsed the END TB Strategy, which prioritizes intensified research and innovation. Here we propose that interrupting transmission become a central focus of that intensified research and innovation.

As we have described herein and in this series, measuring tuberculosis transmission in high-burden settings where each case is not an isolated event represents a significant methodological challenge (Table 1). The dynamic interaction between the tuberculosis and HIV epidemics in many parts of the world further confounds this challenge. Addressing this challenge will require innovative, high-resolution tools, such as geospatial and whole genome sequencing–based analyses of transmission networks [56, 57]. Understanding who transmits to whom, where, and when using new molecular methods will inform the development, deployment, and assessment of precision public health interventions aimed at ending tuberculosis [58]. Countries can and should use such tools to “understand their epidemic” [59] in order to maximize the impact of current interventions and accelerate the application of new tools for the benefit of at-risk populations. For example, the recent decision of Public Health England to institute routine whole genome sequencing for all mycobacterial infections will undoubtedly contribute to advances in our understanding of transmission in the United Kingdom and other low-burden settings [60]. A deeper understanding of tuberculosis transmission may well lead to new interventions to interrupt it, as was the case for HIV with condoms, preexposure prophylaxis, and the dapivirine ring, for example.

**Table 1.** Tuberculosis Transmission Research Needs, Potential Obstacles, and Anticipated Impact and Benefit.

	Research needs	Potential obstacles	Anticipated impact and benefit
**Infectiousness and susceptibility**	Aerobiology: variability in cough aerosol production, role of tidal breathing, airborne survival of Mtb particles	Heterogeneity among patients and limited tools for evaluating aerosol particles	Better tools for preventing aerosol transmission, particularly in nosocomial settings
	Degree of source case infectiousness (eg, for subclinical disease, people with HIV)	Identification of people with subclinical disease prior to onset of symptoms	Understanding of relative contributions from different source cases and ability to target prevention efforts accordingly
	Means to reduce effective contact rates and shared air	Cost and logistics of overhauling congregate facilities; need to engage nonmedical disciplines (eg, engineering, biotechnology)	Reduction of transmission in congregate settings
	Correlates of resistance to tuberculosis infection	Inadequate animal models	Vaccine to prevent infection
**Drivers of transmission**	Local epidemiology and relative contribution of various factors in a given setting	Multidisciplinary approach needed to fully illustrate drivers and catalysts of transmission	Evidence to guide use of limited public health resources for targeted interventions
	Better measures and markers of transmission	TST/IGRA does not differentiate recent from remote infection	Accurate measures of impact of interventions designed to halt transmission
	Community locations of transmission	Difficult to identify epidemiologic links among casual contacts	Identification of congregate areas that may be driving nonhousehold transmission
	Real-time molecular epidemiology and whole genome sequencing to identify linked cases	High cost and technical capacity for molecular epidemiology; transmission occurring from undiagnosed cases	Rapid recognition of outbreaks and potential to intervene and prevent further transmission
**Interventions to halt tuberculosis transmission**	Detailed cross-sectional snapshots of tuberculosis prevalence and transmission at the community level	Difficulty in identifying/diagnosing the tuberculosis cases most associated with transmission	Understanding the sources of tuberculosis transmission in communities (ie, who needs to be evaluated and diagnosed)
	Models and decision aids to prioritize those interventions likely to have greatest impact on transmission in different settings	Assumptions needed for decision making in the absence of complete data	Ability to prioritize those interventions most likely to reduce transmission, given current resource availability
	Clinical trials of interventions designed to halt tuberculosis transmission in populations	Need for preliminary evidence of ability to curb transmission at the population level	Novel evidence-based interventions proven to reduce population-level tuberculosis transmission

Abbreviations: HIV, human immunodeficiency virus; IGRA, interferon-gamma release assay; Mtb, *Mycobacterium tuberculosis*; TST, tuberculin skin test.

The ongoing transmission of tuberculosis in clinics and hospitals is another area of grave concern that directly impacts patients, their families, and the global healthcare workforce. Tuberculosis has become an occupational lung disease for providers from high-burden countries, including frontline community health workers, nurses, medical students, and physicians [61–63]. Tuberculosis as a personal risk associated with a professional choice is unacceptable in the 21st century and must be addressed by national and international professional associations, lest their workers succumb to tuberculosis.

**Figure 1.**
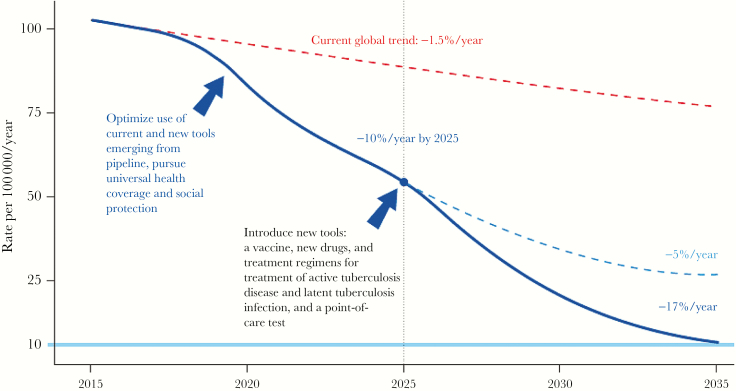
Projected acceleration in the decline of global tuberculosis incidence rates to target levels. From WHO END TB Strategy [62].

Ultimately, both established and new interventions will be required in order to end tuberculosis (Figure 1). Although established interventions such as isoniazid preventive therapy have been quite successful at reducing tuberculosis rates in many settings, several recent studies did not demonstrate a sustained impact for isoniazid preventive therapy in settings where transmission and force of infection is very high [6, 7, 9, 12, 64]. The same may be true for new interventions such as vaccines, which may protect against a single exposure or a low inoculum but be ineffective against repeated, high-dose exposures. The tuberculosis control community should therefore reduce ongoing transmission using all available methods such that any new tools can be effectively deployed. Only by combining the effective use of our existing tools with novel biomedical and social interventions will we be achieve the dramatic declines in incidence necessary to achieve our goals of tuberculosis elimination. And only then will we know that we have reached the end of our research roadmap for tuberculosis transmission science.

In conclusion, continued research in basic discovery, clinical epidemiology, programmatic and population interventions, and cost-effectiveness are required to interrupt transmission and end tuberculosis. We hope that this series furthers that call by highlighting both the gaps and the enormous potential in transmission research. The success of the Global Plan and our collective ability to bend the curve of tuberculosis incidence to zero will require a concerted effort to interrupt transmission today.

## Notes

***Financial support.*** This publication and workshop have been funded in whole or in part with federal funds from the Division of AIDS, National Institute of Allergy and Infectious Diseases (NIAID), US National Institutes of Health (NIH), Department of Health and Human Services under contract number HHSN272201100001G—Research Support Services for the Division of AIDS. Funding support was also provided in part by NIH grants: NIAID R01AI116787 (to D. W. D.) NIAID R01AI089349 (to N. R. G.), NIAID R01AI087465 (to N. R. G.), NIAID K24AI114444 (to N. R. G.), Emory CFAR P30AI050409 (principal investigator: J. W. Curran), and NHLBI T32 HL116271 (principal investigator: D. M. Guidot). The Bill and Melinda Gates Foundation provided funding for participant travel to the workshop. The Centers for Disease Control and Prevention and the South African Medical Research Council co-hosted the corresponding meeting.

***Supplement sponsorship.*** This work is part of a supplement sponsored by the Centers for Disease Control and Prevention and the National Institutes of Health.

***Potential conflicts of interest.*** R. R. is contracted by Kelly Services, which receives federal funds through a government contract with the National Institutes of Health. All other authors declare no potential conflicts. All authors have submitted the ICMJE Form for Disclosure of Potential Conflicts of Interest. Conflicts that the editors consider relevant to the content of the manuscript have been disclosed.
